# Chronic persistent cough in the community: a questionnaire survey

**DOI:** 10.1186/1745-9974-3-5

**Published:** 2007-03-23

**Authors:** Caroline F Everett, Jack A Kastelik, Rachel H Thompson, Alyn H Morice

**Affiliations:** 1 Division of Cardiovascular and Respiratory Studies, University of Hull, Castle Hill Hospital, Cottingham, East Yorkshire, UK

## Abstract

**Background:**

Chronic cough is a common symptom which causes significant levels of morbidity. It is becoming increasingly well characterised by research taking place in specialist cough clinics, where successful treatment rates are high. However, there is a paucity of data regarding the symptom complex of chronic cough in the community. This report details the results of a postal questionnaire survey sent to individuals requesting further information on chronic cough.

**Methods:**

856 chronic cough questionnaires were sent out to members of the public who requested an information sheet following a national UK radio broadcast. Information regarding demography, history of cough, previous treatment and physical, psychological and social effects of the cough was elicited.

**Results:**

373 completed questionnaires were returned. Mean age was 65.3 years (SD 12.0, range 9–88 years). 73% were female and 2% current smokers. Median duration of cough was 6.5 years. 66% had no other coexisting respiratory diagnosis, whilst 24% reported asthma. Of those who responded, 91% had consulted a general practitioner regarding the cough and of them, 85% had been prescribed some sort of treatment. 61% had seen at least one hospital specialist. Commonly reported associated physical symptoms included breathlessness (55%), wheeze (37%), fatigue (72%) and disturbed sleep (70%). Incontinence occurred in 55% of women. Similarly, the majority reported psychological effects such as anger or frustration (83%), anxiety (69%) and depression (55%). 64% felt that the cough interfered with their social life.

**Conclusion:**

Chronic cough causes a high level of morbidity in the community, which results in a correspondingly high rate of healthcare utilisation. Demography and symptomatology seems to be similar to that reported from specialist centres, but successful treatment of the cough was uncommon, despite a high number of medical consultations in both primary and secondary care. If understanding of this debilitating but eminently treatable condition is enhanced, management of chronic cough will improve and many patients will be helped.

## Background

Cough is the commonest symptom for which medical advice is sought [1,2], and sales of over the counter cough syrups alone are worth as much as £92.5 m in the United Kingdom and $328 m in the United States [3]. The majority of cases of cough are acute and self limiting, usually secondary to viral upper respiratory tract infection, however, chronic cough (lasting more than eight weeks) is also a significant problem, with reported prevalence of 10% to 30% [4-6]. Chronic cough is associated with a significant but reversible increase in morbidity, affecting quality of life [7], and would therefore seem to be an important, treatable clinical entity.

Most reports of the aetiology and management of chronic cough originate from specialist cough clinics and therefore reflect the experience of chronic cough in secondary care. Indeed, good data on the prevalence and aetiology of cough in the general population are hard to find. For example, the European Community Respiratory Health Survey targeted a large, unselected group from the general population (18,277 subjects from 16 countries) and included questions on cough [4]. However, only people aged 20 to 48 years were included. Since most series of chronic cough patients show a mean age of 45–58 years [8], it is likely that the European Community Respiratory Health Survey missed a large proportion of people with chronic cough.

This report details the results of a postal questionnaire survey sent to people requesting further information on chronic cough. It provides further information about the demographic and symptomatic profile in a population who consider their cough to be significant.

## Methods

In September 2002 a national UK BBC Radio 4 broadcast took place on chronic cough. This was part of the series "Check Up", which offers medical advice on a different health-related topic each week and is broadcast at 3 pm on a Thursday afternoon. Radio Joint Audience Research Limited (RAJAR) published audience figures for Radio 4 of 9.9 million listeners per week (11.8% share of all radio listeners) for the third quarter of 2002. The BBC estimated that approximately 700,000 people will have listened to this broadcast. Unfortunately, the authors do not have access to specific demographic data on this program's audience.

Of this population, 856 members of the public wrote in with stamped addressed envelopes for an information sheet about chronic cough. The information pack they were sent included a cough questionnaire (see additional file 1), which they were invited to complete and return in a pre-addressed, postage paid envelope, which was also enclosed. The questionnaire used was based on one which is completed by all newly referred patients to the Hull Cough Clinic and completed again when the patients are discharged from the clinic, in order to audit social and demographic factors as well as qualitative measures of response to treatment. It has therefore been completed by over 650 patients, prior to this study, although it has never previously been published. It includes sections asking open questions on demographic details, history of the cough, previous treatment and smoking history, whilst information about the physical, psychological and social effects of the cough is also elicited, using a Likert scale with scores ranging from 1 (never) to 5 (always).

## Results

Of the 856 questionnaires sent out, 373 were completed and returned, giving a response rate of 43.6%. Since not all the respondents answered all questions data is expressed as percentages of the total number who answered a particular question.

### Demographics and history

The mean age of respondents was 65.3 years (standard deviation 12.0, range 9–88 years), with 73% of them being female. 152 (41%) were ex smokers and 8 (2%) were current smokers with a median of 8.0 pack years in these 2 groups (range 0.2–135).

**Figure 1:**
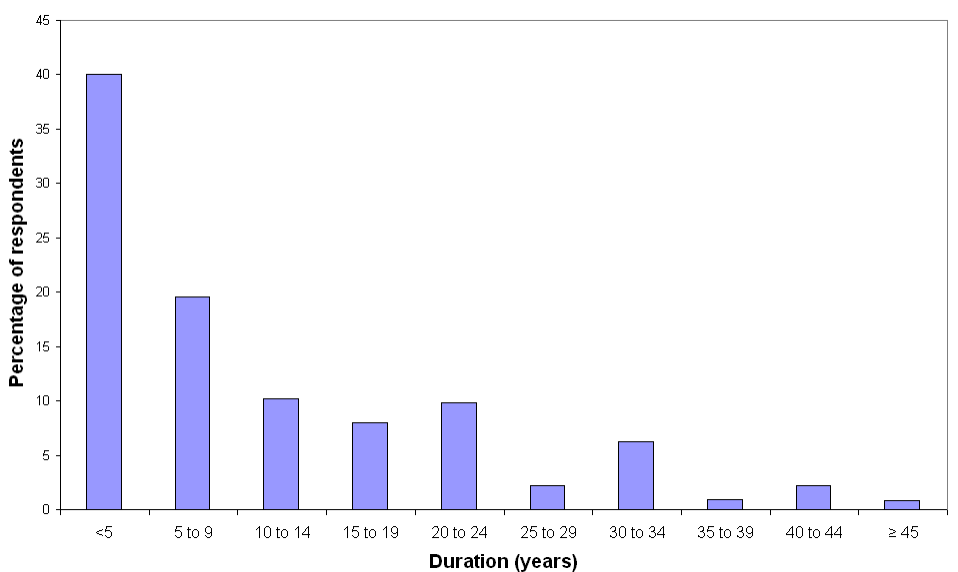
Duration of cough in years.

Duration of cough ranged from 2.5 weeks to 73 years with a skewed distribution. The median duration was 6.5 years, but 40% of respondents had experienced cough for less than 5 years (see figure 1). Severity of cough was rated as moderate by 160 (43%) respondents and as severe or very severe by a further 161 (43%). 39% had 5 bouts of coughing per day or less, 49% coughed between 6 times and 20 times per day and 12% reported bouts of coughing more than 20 times per day. Upper respiratory tract infection preceded the onset of cough in 126 (34%) of subjects.

66% of respondents had no respiratory diagnosis other than cough, whilst 24% reported a diagnosis of asthma, the most commonly reported coexisting respiratory problem. A family history of asthma was reported by 129 (35%) respondents, but only 95 respondents (25%) had one or more first-degree relatives with asthma.

### Previous treatment

Only 34 (9%) of the 373 respondents had not consulted their general practitioner about their cough. Of those who had seen the general practitioner 288 (85%) had been prescribed some sort of treatment for the cough. 226 respondents (61% of the whole sample) had seen one or more hospital specialists regarding cough, with 2 people having seen 5 specialists. Of those who had consulted a specialist, 155 (69%) had seen a respiratory physician.

**Figure 2:**
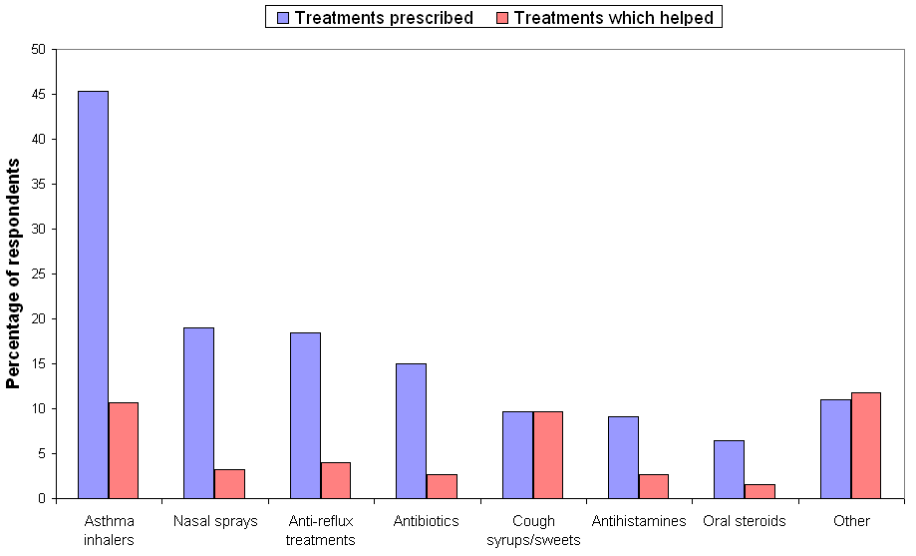
Comparison of treatments prescribed with those perceived to help the cough.

A wide range of medications were reported as having been prescribed for the cough with inhaled steroids and beta 2 agonists being the most common. However, despite the high rates of prescribing, 60% said that their symptoms had not been improved by any treatment. Treatments that were perceived to have helped the cough included inhaled steroids, cough syrup, lozenges and water (see figure 2).

### Physical effects

Cough was commonly associated with other symptoms such as breathlessness (55%), wheeze (37%) and feeling tired or drained (72%). In addition, cough resulted in disturbed sleep in 70%, sore throat in 45% and caused incontinence in 55% of women and 5% of men. Whilst cough syncope was reported by 37 (10%) of subjects, the relatively minor complaint of dizziness on coughing was described by 95 (26%). Most respondents (77%) did not have chest pain as a result of coughing.

62% of respondents complained of sputum production but only 6 (1%) expectorated more than 1 cup of sputum per day with 28 (8%) experiencing haemoptysis at some time. 158 (42%) respondents also had heartburn and 250 (67%) complained of post-nasal drip. In addition, cough affected the voice of 67% of respondents. The majority of respondents (63%) were unable to suppress their cough and activities commonly affected by cough included shopping (33%), housework (34%), climbing stairs (24%) and mealtimes (55%).

### Psychological effects

Psychological effects of the cough were common. 83% of subjects felt anger or frustration as a result of cough and 76% felt out of control of their body. In addition, cough made 69% of responders worry about their health, 55% feel depressed, 80% upset and 76% worried about what others might think. However, only 55 (15%) felt that their cough made them dependent on others with 40% of respondents saying that the cough seldom or never significantly altered their lives.

### Social effects

64% of respondents felt that cough affected their social life. Many described altering their behaviour such as how often they go to the cinema/bingo (39%) or restaurants (34%) and avoiding things that trigger the cough (60%). For example, 71 respondents (19%) said their cough affected how often they visited friends or relatives. Other areas affected by cough included phone calls (81%) and hobbies (45%).

Although only 169 (45%) of the respondents were in employment, 53% of them felt it was affected by the cough. 5 of the 20 smokers (25%) said that cough affected how many cigarettes they smoked.

## Discussion

In the past it has been difficult to provide accurate data on the epidemiology of chronic cough. A number of questionnaire surveys have tried to evaluate the prevalence of respiratory symptoms [4,6], but they were not designed specifically to assess chronic cough and its effects on quality of life. Many early studies used Medical Research Council criteria to assess prevalence of chronic bronchitis in a population [4-6]. For this reason the information they provide is not always applicable to the population suffering from clinically significant chronic cough. More recently, however, a large community cross-sectional survey has confirmed the significant prevalence and female preponderance of chronic cough in the community [9].

Until recently understanding of the effects of chronic cough on health status has been limited, although work on cough specific quality of life tools is now starting to provide us with measurable health outcome data [7,10]. However, these tools have only been used so far in patients attending specialist cough centres, reflecting the experience of chronic cough in secondary and tertiary care. The present study reports on the demographic data as well as the effects of chronic cough on physical, social and psychological aspects of health of a large group of self selected patients with chronic cough, recruited from the general Radio 4 listening public. However although much of the data was collected in numeric form (on a Likert scale), in order to gain some indication of severity, the results must be regarded as qualitative, rather than quantitative, as this questionnaire has not previously been formally validated.

This study population evidently can not be said to represent *all *subjects in the community with cough, due to the usual types of selection bias associated with this type of study. Self selection of questionnaire respondents and factors such as time of day, mode and network of the broadcast mean that the demographics of listeners to the radio broadcast cannot be expected to be wholly representative of the general population. However, the large estimated audience of 700,000 suggests that they are drawn from a wider group than the population usually seen in a specialist cough clinic and the fact that they have responded to an unsolicited questionnaire suggests that these data represent a profile of a clinically relevant group suffering from a troublesome chronic cough.

Notable similarities exist between the demography of our study population and that described in previous reports from secondary care. For example, the high proportion of females (73%) is similar to that reported in the recent literature, with published series from various specialist clinics consisting of between 55% and 78% females [11]. In clinical practice this marked gender difference is thought to be related to the observation that cough reflex sensitivity is heightened in both female healthy volunteers [12,13] and in female chronic cough patients [14], when compared with their male counterparts. However, although the gender distribution of chronic cough in our community-based sample corresponds well with observations in secondary and tertiary care, the mean age of 65.4 years in our population was somewhat higher than the range of mean ages (45 to 58 years) quoted in the literature [8]. It is impossible to tell whether these findings were related either wholly or in part to selection and reporting bias or whether other factors, such as increased cough sensitivity in women or limitation of access to tertiary referral cough clinics are also responsible. However, RAJAR audience profiling figures for the timeslot in which this radio broadcast was made suggest that the listeners were 54% female with a mean age of 56. This would suggest that the demographics of our study population may not be entirely due to the age and gender profile of the audience.

Past experience reveals that although smoking is known to be associated with a dose related increase in reported cough [4], in practice smokers rarely seek medical advice for cough [14]. This presumably is because they do not perceive the cough to require medical attention, or they erroneously ascribe their chronic cough to smoking and is consistent with the very low proportion of current smokers (2%) who presented in this survey.

This survey confirms that chronic cough is poorly treated in the studied population. Despite a high rate of medical consultations and of prescribing the median duration of cough was still 6.5 years. 24% of respondents claimed to have a pre-existing diagnosis of asthma and 32% had been prescribed either oral or inhaled corticosteroids at some point, but only 9% of respondents reported that these treatments had helped at all. This may be due, at least in part, to the self selected nature of the population as individuals who had gained good effect from prescribed medications might be less likely to respond to the questionnaire; however other explanations are also possible. For example, the accuracy of the diagnoses of asthma cannot be confirmed as we have no information regarding who the diagnosis was made by, or the grounds on which it was made. Even if a correct diagnosis of asthma has been made, this does not rule out the presence of some other additional cause of cough such as reflux disease, which would not improve with steroid treatment. In addition, we have no information regarding the dose or duration of treatment which, if inadequate, might contribute to the likelihood of treatment failure.

Cough syrups, lozenges and water, however, ranked highly as treatments that were alleged to help the cough, outranking many prescribed treatments such as beta-2 agonists and nasal steroids. Only 10% of respondents reported that cough syrups and sweets had been prescribed but, when asked which treatments (prescribed or self-medicated) had helped the cough, approximately 10% of respondents stated that syrups or sweets had helped and 12% gave answers such as cold water, chewing gum, alcohol, etc, which were grouped in the "Other" category in this report. This may simply reflect the fact that these remedies are much more freely available to the public than prescription medications, but it is interesting to note their perceived efficacy especially since most over the counter cough remedies rely on similar demulcent and non-pharmacological strategies which may have previously been ascribed to "placebo effect" [15]. Their reported efficacy in this study and burgeoning over the counter sales casts doubt on reports that they do not significantly improve cough symptoms.

The impact of chronic cough on health status is varied, ranging from minimal in some patients to a disabling symptom in others. However, the reasons which lead patients to seek advice are complex and poorly understood [16]. Work developing cough specific quality of life measures in secondary care has revealed effects of chronic cough in physical, psychological and social health domains [7,10], which are consistent with our community-based data. For example, in the psychological domain, feelings such as anger, frustration, anxiety and depression were reported by a majority of questionnaire respondents. Similarly, our results show that cough affected social life in two thirds of subjects, leading many of them to alter their behaviour, often avoiding situations and places which might trigger the cough or where they might be embarrassed by the cough. Cough related morbidity in terms of physical symptoms was also varied with cough associated breathlessness, sore throat, fatigue and sleep disturbance being prominent. These extensive and potentially significant effects of cough on health status highlight the importance of a detailed history of associated symptoms and concerns when assessing a patient with chronic cough.

Although this questionnaire was not designed to be a diagnostic tool, there were several questions which may give clues as to the possible underlying causes of the cough. Previous work suggests that gastroesophageal disease, asthma and rhinitis are the most common causes of chronic cough [3]. In this survey the majority of respondents reported one or more symptoms which might be suggestive of these diagnoses, such as heartburn, wheeze and post-nasal drip. Although this data is far from sufficient to make any conclusions about the causes of the reported cough, it is interesting to note that only 13% of people had none of the aforementioned symptoms which, if reported in a cough clinic, might lead to further investigation or treatment of these common aetiological factors. Other symptoms suggestive of more serious pulmonary pathology, such as expectoration of more than 1 cup of sputum per day and haemoptysis had a reassuringly low prevalence (1% and 8% respectively). Vocal symptoms, however, were very common. This, coupled with the high incidence of cough on phonation, for example on the telephone, might lead a clinician to consider a possible diagnosis of laryngopharyngeal reflux, a diagnosis which is often under-recognised in chronic cough patients. This syndrome of laryngeal irritation is caused by supra-oesophageal reflux of gastric juices and has different characteristics to gastroesophageal reflux related to oesophagitis [17]. At present the prevalence of laryngopharyngeal reflux as a cause of chronic cough is not known.

The presumption that chronic cough represents a significant burden on NHS resources and especially on primary care services, is supported by the observation that 91% of respondents to this survey had consulted a general practitioner about the cough and 60% had seen at least one hospital specialist. However, the fact that only 40% of respondents had found a treatment that helped indicates that it is sub-optimally managed in this population, since several series of systematic management show treatment success rates in excess of 90% [11]. Although we must acknowledge that subjects with unresolved and on-going troublesome cough would be more likely to seek information and therefore answer this questionnaire than those whose cough had resolved with treatment, the fact that these individuals had sought medical advice from several sources, without success is undeniable. We suggest the main reason underlying this failure is the poor recognition in both primary and secondary care of the aetiology of chronic cough [18]. Since the morbidity of the physical, psychological and social symptoms associated with chronic cough is high and simple treatments are often highly successful it should be possible to manage this unmet need more effectively.

## Conclusion

In conclusion, we have shown that chronic cough causes a high level of morbidity in affected individuals, which results in a correspondingly high rate of healthcare utilisation by these individuals. In the authors' opinion, chronic cough is currently poorly diagnosed and managed outside of specialist cough clinics, mainly due to a widespread lack of knowledge of the aetiology of this debilitating, but eminently treatable symptom. If understanding is enhanced, management of chronic cough may improve and many patients will be helped.

## Authors' contributions

CFE collated and analysed data from the returned questionnaires and drafted the manuscript. JAK and RHT both participated in design of the study and of the study questionnaire. RHT also collected and collated data from the questionnaires. AHM conceived of the study, participated in its design and coordination, took part in the initial radio broadcast and helped to draft the manuscript. All the authors read and approved the final manuscript.

## Supplementary Material

Additional File 1 Chronic cough questionnaire. Blank template of the postal questionnaire survey which was sent to people requesting further information on chronic cough, following the Radio 4 broadcast. Click here for file
