# Supplementary material for: Chronic persistent cough in the community: a questionnaire survey
Source: Cough. 2007 Mar 23;3:5. doi: 10.1186/1745-9974-3-5 (PMC1847685; doi:10.1186/1745-9974-3-5)
Supplement: Additional File 1 — Chronic cough questionnaire. Blank template of the postal questionnaire survey which was sent to people requesting further information on chronic cough, following the Radio 4 broadcast. [file 1745-9974-3-5-S1.doc]

Chronic Cough Questionnaire

###### What is the questionnaire about?

This is a questionnaire for people who suffer with a persisitent or chronic cough. It is designed to assess the impact of their cough on their physical, psychological and social well being. The questions are referring to how your cough affects you generally, ie on average how does your cough affect you, therefore not when you are at your best or worse. It is best to refer to your symptoms within the last 2 weeks.

###### Who should complete this questionnaire?

The questions should be answered by the person named on the envelope. If that person needs help to complete the questionnaire the answers should still be given from his/her point of view-not the point of view of the person who is helping.

###### How to complete this questionnaire

To reduce the time it takes to read the questionnaire please tick clearly inside the boxes using a black or blue pen. The questions can be answered by simply ticking the answer that applies to you. Tick only one box per question, unless otherwise stated.

## Example

## Do you sometimes feel breathless? Tick one only

Never 1

Seldom 2

Sometimes 3

Often 4

Always ^5^

NA 0

**Questions or help**

If you have any queries about the questionnaire please call Castle Hill Hospital on 01482 624009. The line will be open between 9:00am and 5:00pm Monday to Friday and will be connected to an answerphone at other times

# Part A: Your details

**A1** NAME

(Please do not complete this question if you would rather remain anonymous)

**A2** DATE OF BIRTH ___/___/___

**A3** POST CODE

**A4** AGE:

**A4** GENDER: Male Female

# **Part B: About your cough**

Unbearable symptoms

**B1** Duration of cough_____________

10 -

9 -

8 -

7 -

6 -

5 -

4 -

3 -

2 -

1 -

0 -

**B2** Description of cough

**B2.1** Severity of cough:

Indicate on the adjacent scale how severe you feel your cough is. 0 is no cough at all and 10 is a continuous most disturbing cough (unbearable symptoms).

**B3** Was cough preceded by chest/ upper airway infection Yes No

No cough

### Part C: Your previous treatment

**C1** Have you visited your G.P regarding your chronic cough?

Yes No

If yes, did your doctors prescribe any medication?

Yes No

If yes, what was the medication/s called?

Does any treatment help your cough?

Yes No

If yes, what

**C2** Have you seen a hospital specialist regarding your chronic cough?

Yes No

If yes, how many consultants have you seen?

From which departments?

Respiratory/chest

ENT

Gastroenterology

Other

**C3** Have you been diagnosed with any breathing problems other than chronic cough?

Yes No

If yes tick the appropriate condition/s:

Asthma Emphysema Bronchiectasis COPD

Other (specify)

**C4** Does anyone one in your family suffer with asthma? Yes No

If yes whom:

# **Part D: Smoking history**

**D1** Please α the box

1. I have never smoked
2. I smoke at the moment

If yes, how many cigarettes a day? ___________

For how many years? ____________

(c) I don’t smoke now but have in the past

If yes, how many cigarettes a day? ___________

For how many years? ____________

How long ago did you stop smoking? ______________

### Part E: General

# Please tick the appropriate box that on average best describes your cough

## E1 How many bouts of coughing a day do you

## generally suffer with? Tick one only

Hardly ever – 0-1 1

2-5 2

6-10 3

11-20 4

All the time - 20^+ 5^

**E2** How would you rate your cough? Tick one only

Very mild 1

Mild 2

Moderate 3

Severe 4

Very severe 5

#### Part F: Physical effects

# Please tick the appropriate box that on average best describes the physical symptoms, if any, associated with your cough

## F1 Do you sometimes feel breathless? Tick one only

Never 1

Seldom 2

Sometimes 3

Often 4

Always ^5^

NA 0

## F2 Do you ever feel wheezy? Tick one only

Never 1

Seldom 2

Sometimes 3

Often 4

Always ^5^

NA 0

## F3 Do you ever get chest pain due to your cough? Tick one only

Never 1

Seldom 2

Sometimes 3

Often 4

Always ^5^

NA 0

**Still thinking about the physical symptoms associated with your cough……….**

## F4 Do you ever get pain under your ribs due to your cough? Tick one only

Never 1

Seldom 2

Sometimes 3

Often 4

Always ^5^

NA 0

## F5 Does your cough make you faint? Tick one only

Never 1

Seldom 2

Sometimes 3

Often 4

Always ^5^

NA 0

## F6 Does your cough make you feel dizzy? Tick one only

Never 1

Seldom 2

Sometimes 3

Often 4

Always ^5^

NA 0

## Still thinking about the physical symptoms associated with your cough……….

## F7 Do you feel drained or tired due to your cough? Tick one only

Never 1

Seldom 2

Sometimes 3

Often 4

Always ^5^

NA 0

## F8 Does your cough make your throat sore? Tick one only

Never 1

Seldom 2

Sometimes 3

Often 4

Always ^5^

NA 0

## F9 Does your cough affect your voice? Tick one only

Never 1

Seldom 2

Sometimes 3

Often 4

Always ^5^

NA 0

**Still thinking about the physical symptoms associated with your cough……….**

## F10 Are you able to speak when you cough? Tick one only

Never 1

Seldom 2

Sometimes 3

Often 4

Always ^5^

NA 0

## F11 Do you ever loose control of your bladder or bowels due to your cough?

## Tick one only

Never 1

Seldom 2

Sometimes 3

Often 4

Always ^5^

NA 0

## F12 Do you cough up phelgm? Tick one only

Never 1

Seldom 2

Sometimes 3

Often 4

Always ^5^

NA

If yes, is the quantity greater than one cup a day? Yes No

#####

## F13 Do you cough up blood? Tick one only

Never 1

Seldom 2

Sometimes 3

Often 4

Always ^5^

NA 0

## F14 Are you able to suppress your cough? Tick one only

Never 5

Seldom 4

Sometimes 3

Often 2

Always ^1^

NA 0

## F15 Does your cough affect your sleep? Tick one only

Never 1

Seldom 2

Sometimes 3

Often 4

Always ^5^

NA 0

## Still thinking about the physical symptoms associated with your cough……….

## F16 Does your cough affect you doing your shopping? Tick one only

Never 1

Seldom 2

Sometimes 3

Often 4

Always ^5^

NA 0

## F17 Does your cough affect you doing housework/cleaning? Tick one only

Never 1

Seldom 2

Sometimes 3

Often 4

Always ^5^

NA 0

## F18 Does your cough affect you climbing stairs? Tick one only

Never 1

Seldom 2

Sometimes 3

Often 4

Always ^5^

NA 0

**Still thinking about the physical symptoms associated with your cough……….**

## F19 Does your cough interfere with meals? Tick one only

Never 1

Seldom 2

Sometimes 3

Often 4

Always ^5^

NA 0

**F20** Do you suffer with heartburn or indigestion? Yes No

**F21** Do you suffer with a stuffy nose or mucus trickling down the back of your throat (post nasal drip)?

Yes No

### Part G: Other effects

# Please tick the appropriate box that on average best describes the psychological effects if any, associated with your cough

## G1 Does your cough make you feel angry or frustrated? Tick one only

Never 1

Seldom 2

Sometimes 3

Often 4

Always ^5^

NA 0

**Still thinking about the other effects associated with your cough……….**

## G2 Does your cough make you feel that your not in control of your body?

## Tick one only

Never 1

Seldom 2

Sometimes 3

Often 4

Always ^5^

NA 0

## G3 Does your cough make you worry about your health? Tick one only

Never 1

Seldom 2

Sometimes 3

Often 4

Always ^5^

NA 0

## G4 Does your cough make you feel depressed? Tick one only

Never 1

Seldom 2

Sometimes 3

Often 4

Always ^5^

NA 0

**Still thinking about the other affects associated with your cough……….**

## G5 Does your cough upset you? Tick one only

Never 1

Seldom 2

Sometimes 3

Often 4

Always ^5^

NA 0

## G6 Does your cough makeyou feel dependant on other people? Tick one only

Never 1

Seldom 2

Sometimes 3

Often 4

Always ^5^

NA 0

## G7 Do you worry about what others may think about your cough? Tick one only

Never 1

Seldom 2

Sometimes 3

Often 4

Always ^5^

NA 0

### Part H: Social effects

Please tick the appropriate box that on average best describes the social effects if any, associated with your cough

## H1 Does your cough affect your social life? Tick one only

Never 1

Seldom 2

Sometimes 3

Often 4

Always ^5^

NA 0

## H2 Does your cough affect the frequency which you go to the cinema, bingo etc…?

Never 1

Seldom 2

Sometimes 3

Often 4

Always ^5^

NA 0

## H3 Does your cough affect the frequency which you go to restaurants?

## Tick one only

Never 1

Seldom 2

Sometimes 3

Often 4

Always ^5^

NA 0

**Still thinking about the social effects associated with your cough……….**

## H4 Do you avoid things that bring on your cough? Tick one only

Never 1

Seldom 2

Sometimes 3

Often 4

Always ^5^

NA 0

## H5 Does your cough affect the frequency which you visit friends or relatives?

## Tick one only

Never 1

Seldom 2

Sometimes 3

Often 4

Always ^5^

NA 0

## H6 Does cough affect telephone calls? Tick one only

Never 1

Seldom 2

Sometimes 3

Often 4

Always ^5^

NA 0

**Still thinking about the social effects associated with your cough……….**

## H7 Does cough interfere with your hobbies? Tick one only

Never 1

Seldom 2

Sometimes 3

Often 4

Always ^5^

NA 0

## H8 Does your cough affect your job? Tick one only

Never 1

Seldom 2

Sometimes 3

Often 4

Always ^5^

NA 0

## H9 Does your cough affect how many cigarettes you smoke? Tick one only

Never 1

Seldom 2

Sometimes 3

Often 4

Always ^5^

NA 0

**Overall, taking into account the physical, other effects and social changes your cough has caused…………**

## I1 Has your cough significantly altered your life? Tick one only

Never 1

Seldom 2

Sometimes 3

Often 4

Always ^5^

NA 0

**THANK YOU FOR YOUR TIME AND EFFORT**

###### Please check that you have answered all the questions that you wish to answer

**Please post this questionnaire in the envelope provided. No stamp is needed.**

Academic Medicine, Castle Hill Hospital, Castle Road, Cottingham, East Yorkshire, HU16 5JQ, UK

**Tel: +44 (0) 1482 624067 Fax: +44 (0) 1482 624068**
